# Determinants of Quality of Life among Adolescents in the Małopolska Region, Poland

**DOI:** 10.3390/ijerph19148616

**Published:** 2022-07-15

**Authors:** Agnieszka Magiera, Agnieszka Pac

**Affiliations:** Department of Epidemiology and Preventive Medicine, Faculty of Medicine, Jagiellonian University Medical College, Kopernika 7a, 31-034 Kraków, Poland; agnieszka.pac@uj.edu.pl

**Keywords:** adolescents, quality of life, KIDSCREEN-52

## Abstract

Knowledge about predictors associated with quality of life (QoL) in adolescents is important for public health. The aim of the study was to indicate determinants of the different dimensions of QoL in the fields of demographic, socio-economic factors, general health and lifestyle in a sample of Polish adolescents. The cross-sectional study was carried out in a southern region of Poland among 804 schoolchildren from junior high schools and upper secondary schools. The quality of life was measured using the Polish version of the KIDSCREEN-52 questionnaire. The author’s questionnaire concerning determinants of the adolescents’ quality of life was also used. In the analysis of the quality of life, standardized results on the European population (T-score) and categorization on the low, average and high quality of life were taken into account. Defining the possible determinants of the adolescents’ quality of life was made by the multivariate logistic regression models. The highest prevalence of low QoL was observed in the school environment (53.4%) and the psychological well-being (51.6%) dimensions of the KIDSCREEN-52. The factors that increased the risk of the low assessment of the quality of life were, inter alia, female sex for physical well-being, psychological well-being, self-perception, autonomy, parent relation and home life, and financial resources, higher school year for physical well-being, psychological well-being, moods and emotions (2nd grade of upper secondary school only), self-perception, social support and peers, and school environment, and dissatisfaction in appearance for physical well-being, psychological well-being, moods and emotions, self-perception, school environment, social acceptance and bullying.

## 1. Introduction

The World Health Organization (WHO) defined quality of life (QoL) as “perception by the entity’s position in life, in the context of culture and value systems in which they live and the relation to its objectives, expectations, standards and interests” [1]. According to the WHO’s definition, QoL is interpreted as a multidimensional construct.

The definition of QoL among children and adolescents is not widespread in studies, compared with the definition for adults. More often than not, the QoL of children and adolescents is related to the definitions intended for adults, whereas studies on the QoL in youth are conducted using specific tools for adolescents [2,3,4]. Additionally, in these studies, little attention is devoted to associations between QoL and determinants of QoL in this age group [5].

Knowledge about predictors associated with QoL in children and adolescents is important for public health. Recognition of factors that affect QoL may help identify those who need support or professional help [6,7]. The studies on QoL and the determinants of QoL among children and adolescents can provide a better understanding of young people’s needs and help in the early identification of low QoL [7,8] and the development of preventive actions.

The assessment of QoL should be related to the most important domains and problems faced by adolescents [5]. The significant determinants of quality of life among youth are: feeling good, satisfaction with health status, self-satisfaction, positive self-image, positive self-attitude, attitude towards each other, close relationships with family and peers, and forming new relationships [9].

Generally, previous international studies on the determinants of QoL in adolescents showed demographic predictors of low QoL, such as female sex and age. The majority of studies identified that girls assessed QoL lower than boys [10,11,12] and older adolescents had poorer QoL than the younger ones [13,14]. Other studies also demonstrated that socio-economic variables such as lower father’s education, low material standing of the family [15,16], and lack of comfort at home [17] are significant predictors of low QoL in teenagers. Additionally, the way teenagers perceive their own bodies and physical appearance may affect their perceived health and QoL [7], so adolescents who were dissatisfied with their bodies and appearance were more likely to have poorer QoL [11]. Previous studies also identified decreased physical activity as a significant factor associated with poor QoL in adolescents [18]. Similarly, teenagers who declared a lack of relationships with peers and friends were more likely to experience low QoL and emotional as well as social problems [19].

Based on earlier studies in the literature, we focused on four groups of variables: demographic, socio-economic factors, general health, and lifestyle as predictor variables of QoL among adolescents. The aim of the present study was to indicate determinants of the different dimensions of quality of life of adolescents in the fields of demographic and socio-economic factors, general health and lifestyle in a sample of Polish adolescents.

## 2. Materials and Methods

### 2.1. Procedure and Sample

The cross-sectional study was carried out in a southern region of Poland (the Małopolska region) among schoolchildren from 10 schools (5 junior high schools, 5 upper secondary schools) between May 2016 and June 2017. We aimed to assess students’ quality of life every two years during the adolescence period. So, we decided to conduct the study in junior high schools (education period—3 years; age 13–15 years) and upper secondary school (education period—3 years; age 16–18 years). We have chosen to study every second grade, i.e., students in 1st and 3rd grade of junior high schools, and then the 2nd grade of upper secondary schools (all types of upper secondary schools) was invited to participate in this study. Two-stage sampling was applied to select the participating schools. First, five counties from the Małopolska region were randomly selected, and then one junior high school and one upper secondary school from each county were randomly selected based on the Local Education Authority registry. The school head teachers agreed to participate in the study. Parents/caregivers (during periodical teacher–parent meetings) and schoolchildren were given information about aims of the study and study protocol. A signed written informed consent was obtained from the parents/fosterers or adolescents aged 18 years. The only exclusion criterion was the lack of agreement to participate of parents/caregivers or adolescents aged 18 years.

Adolescents filled in the questionnaires in classrooms during lesson time in the presence of the investigator, who could provide assistance when needed. 

Out of a total number of 1664 adolescents from the enrolled schools, 554 (33.3%) did not return the written consent from their parents/caregivers, 116 (7%) were absent at school on the day the study was performed and 135 (8.1%) did not want to participate. Hence, 859 adolescents took part in the study, for an overall response rate of 51.6%.

This analysis was based on the data from 804 adolescents; 30 questionnaires were excluded because of missing data in the KIDSCREEN-52 questionnaire, and 25 were excluded because of missing data in other parts of the questionnaire. 

The study was approved by the Jagiellonian University Bioethics Committee (approval number: 122.6120.6.2016).

### 2.2. Instruments

The Polish version of the KIDSCREEN-52 questionnaire was used to measure the quality of life (QoL). The KIDSCREEN-52 questionnaire is an international, validated instrument for children and young people aged 8–18 years. It can be used for cross-cultural comparisons. The 52-item version of KIDSCREEN covers 10 dimensions:

1. Physical Well-Being (5 items)—the level of the child’s/adolescent’s physical activity, energy, and fitness as well as capacity for lively or energetic play. The Cronbach’s alpha for this subscale is 0.810 [20].
2. Psychological Well-Being (6 items)—the psychological well-being of the child/adolescent including positive emotions and satisfaction with life. The Cronbach’s alpha for this subscale is 0.905 [20].
3. Moods and Emotions (7 items)—the child’s/adolescent’s experience of depressive moods, emotions and stressful feelings. The Cronbach’s alpha for this subscale is 0.879 [20].
4. Self-Perception (5 items)—the child’s/adolescent’s perception of self, security and satisfaction about himself/herself as well as his/her appearance. The Cronbach’s alpha for this subscale is 0.806 [20].
5. Autonomy (5 items)—the opportunity given to a child or adolescent to create his/her social and leisure time; the child’s/adolescent’s freedom of choice, self-sufficiency, and independence activities. The Cronbach’s alpha for this subscale is 0.854 [20].
6. Parent Relation and Home Life (6 items)—the relationship between the parents and the atmosphere in the child’s/adolescent’s home. The Cronbach’s alpha for this subscale is 0.902 [20].
7. Financial Resources (3 items)—the child’s/adolescent’s feelings about having enough financial resources to allow him/her to live a lifestyle which is comparable to other children/adolescents. The Cronbach’s alpha for this subscale is 0.908 [20].
8. Social Support and Peers (6 items)—the quality of interactions between the child/adolescent and peers as well as their perceived support. The Cronbach’s alpha for this subscale is 0.866 [20].
9. School Environment (3 items)—the child’s/adolescent’s perception of his/her cognitive capacity, learning and concentration, and his/her feelings about school. The Cronbach’s alpha for this subscale is 0.866 [20].
10. Social Acceptance and Bullying (3 items)—the feeling of being rejected by others as well as the feeling of anxiety towards peers. The Cronbach’s alpha for this subscale is 0.807 [20].

The response range for each KIDSCREEN-52 item is based on a 5-point Likert scale. The scale indicates the frequency of certain behaviors or feelings (1 = never, 2 = seldom, 3 = quite often, 4 = very often, 5 = always) or the intensity of an attitude (1 = not at all, 2 = slightly, 3 = moderately, 4 = very, 5 = extremely). The time frame refers to the previous week [21]. The Polish version of the child KIDSCREEN-52 has shown satisfactory validity as well as reliability to be used in the Polish population, with Cronbach alphas in the range from 0.806 to 0.908 [20]. In this study, Cronbach’s alphas ranged from 0.777 to 0.923, suggesting good internal consistency of the questionnaire.

The quality-of-life scores in each dimension were transformed into T-scores based on the guidelines developed by the authors of the scale for the European reference population of adolescents aged 12–18 years from the KIDSCREEN project [21]. Later, adolescents’ quality of life in each dimension (based on T-score) was categorized as low, average or high according to sex- and age-specific cut-point values based on the European standards for adolescents aged 12–18 years from the KIDSCREEN project. Low quality of life was defined as a score lower than the 25th percentile of a distribution, while high quality of life was defined as higher than the 75th percentile of a distribution.

### 2.3. Other Characteristics

Based on the information obtained from the questionnaire, four groups of variables were identified as potentially affecting adolescent’s QoL: demographic characteristics, socio-economic factors, health and satisfaction with health status as well as own appearance, and lifestyle. 

The first part of the questionnaire recorded demographic details, such as sex and school year. The second part was related to general health details, such as satisfaction with health status, satisfaction with appearance, and absence at school due to illness episodes. The next part consisted of socio-economic items such as father’s/caregiver’s education, receiving pocket money from parents/caregivers, good material standing of the family, good relations with at least one parent/caregiver, and feeling comfortable at home. 

The last questions were related to adolescents’ lifestyle, such as time for social media (instant messengers, social networks) per week, time for physical activity per week (besides physical education classes), and spending free time with friends outside of school.

### 2.4. Statistical Analysis

The data have been presented as means and SD (standard deviation) for normally distributed or medians and quartiles for non-normally distributed quantitative variables, and frequencies and percentages for qualitative ones. Multivariable logistic regression models were used to find factors related to the low QoL. The low QoL was a dependent variable in these models (a separate model was created for each dimension of QoL). Then, variables that had an impact on the low QoL in each dimension were selected and multivariable logistic regression model was carried out.

The analyses were performed using IBM SPSS Statistics 24, and results with *p* < 0.05 were considered as statistically significant.

## 3. Results

### 3.1. Characteristics of the Sample 

A total of 804 adolescents were included in this analysis—393 (48.9%) girls and 411 (51.1%) boys aged 12–18 years. The average age of the participants was 16.1 years (SD 1.69). More than half of adolescents were from the 2nd grade of upper secondary school students (58%).

Most of the adolescents declared good family material standing (85.7%) and receiving pocket money (90.6%). Almost 85% of the respondents declared being satisfied with their health status, whereas almost 70% of the adolescents declared being satisfied with their appearance.

More than 19% of the adolescents declared more than 3 h of physical activity per week (besides physical education classes). Almost 88% of the adolescents reported that they spend their free time with friends outside of school. Table 1 presents the detailed sample characteristics.

### 3.2. Quality of Life

Based on data transformation into T-score values, we observed that quality of life (QoL) scored the lowest in school environment dimension (median = 42.35). Additionally, the psychological well-being dimension was assessed as low (median = 43.25) (see Table 2).

**Figure 1:**
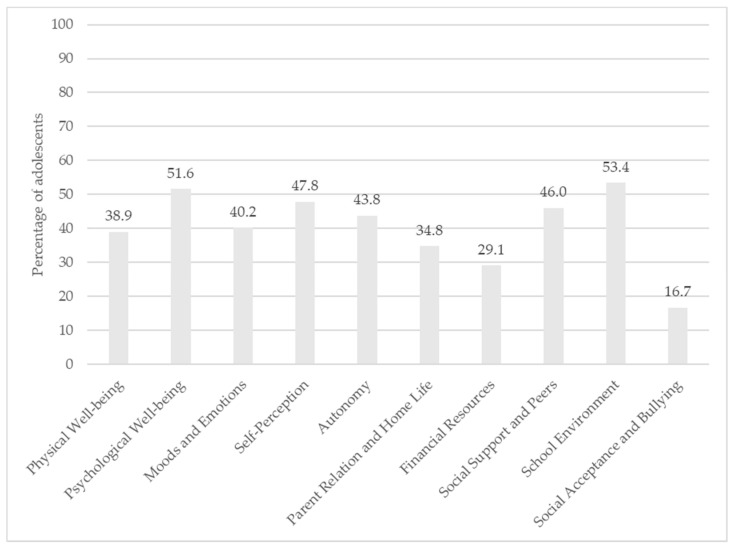
The prevalence of low quality of life in adolescents in 10 KIDSCREEN-52 dimensions (in percentages).

Furthermore, the QoL across the 10 KIDSCREEN-52 dimensions was divided into low, middle and high groups based on the European standards for sex and age from the KIDSCREEN project. Figure 1 shows the prevalence of low QoL in the whole group. After this categorization, the highest prevalence of low QoL was observed in school environment dimension—53.4% of the respondents—and the psychological well-being dimension—51.6% of the respondents (see Figure 1).

In turn, the lowest prevalence of high QoL was observed in school environment dimension—only 5.1% of the adolescents—and for moods and emotions dimension (13.4% of participants assessed their QoL as high). Among all the KIDSCREEN-52 dimensions, the social acceptance and bullying dimension was the highest assessed—almost 17% of adolescents declared low QoL.

### 3.3. Predictors of Low Quality of Life of Adolescents

Then, we wanted to indicate potential determinants of the QoL among adolescents in the fields of demographic and socio-economic factors, general health, and lifestyle, so we applied the multivariate logistic regression model with the low QoL as the dependent variable (see Table 3). 

In the field of demographic factors, sex and school year were significantly associated with low QoL of the respondents. Girls had higher odds of low QoL than boys in the dimensions physical well-being, psychological well-being, self-perception, autonomy, parent relation and home life and financial resources (see Table 3). There were also significantly higher odds of low QoL for the school year; being a student of the 3rd grade of junior high school or the 3rd grade of upper secondary school was associated with lower QoL.

Among the variables involved in health status, being dissatisfied with health status was related to higher odds of low QoL in the dimensions physical well-being (OR = 1.69; 95% CI: 1.02–2.80), psychological well-being (OR = 2.07; 95% CI: 1.22–3.63) and parent relations and home life (OR = 1.72; 95% CI: 1.05–2.82). Being dissatisfied with appearance was associated with lower QoL in the majority of KIDSCREEN-52 dimensions. Adolescents who were dissatisfied with their appearance had higher odds of low QoL, ranging from 1.80 (physical well-being) to 5.22 times (self-perception). A significant relationship between absence at school due to illness episodes and odds of low QoL was found only in the domains of physical well-being and school environment. In the physical well-being dimension, absence at school was often a predictor of a young person’s low QoL (OR = 2.47; 95% CI: 1.48–4.13). In the school environment dimension, the respondents who declared absence at school due to illness episodes often had nearly 1.8 times higher odds of low QoL (OR = 1.78; 95% CI: 1.10–2.89) (see Table 3). 

Among the examined adolescents, we observed that important determinants of low QoL were those related to socio-economic factors. Father’s/caregiver’s education was a predictor of low QoL only in the self-perception domain. Primary/vocational education significantly increased the odds of low QoL, almost 1.5 times (OR = 1.49; 95% CI: 1.06–2.10). The adolescents who declared not receiving pocket money had almost 2 times higher odds of low QoL in moods and emotions (OR = 1.82; 95% CI: 1.01–3.28), more than 2 times higher in autonomy (OR = 2.28; 95% CI: 1.24–4.19) and social support and peers (OR = 2.25; 95% CI: 1.27–3.98), and nearly 6.5 times higher in the financial resource dimension (OR = 6.43; 95% CI: 3.42–12.11). Poor material standing of the family was a risk factor of low QoL in dimensions related to youths’ psychological well-being, moods and emotions, autonomy and financial resources in a range from 1.86 (moods and emotions) to 5.34 (financial resources). Lack of good relations with at least one parent/caregiver was a predictor of low QoL in five out of ten KIDSCREEN-52 dimensions. This was the factor that increased the odds of low QoL 2 times in financial resources (OR = 2.00; 95% CI: 1.01–3.94) and almost 2 times in social support and peers (OR = 1.98; 95% CI: 1.06–3.71). Moreover, adolescents were more likely to experience low QoL more than 2 times in psychological well-being (OR = 2.37; 95% CI: 1.13–4.97) and moods and emotions (OR = 2.10; 95% CI: 1.09–4.04) dimensions. However, in the parent relation and home life domains, the lack of good relations was a risk factor of low QoL by more than 5.5 times (OR: 5.67; 95% CI: 2.73–11.81). Respondents who reported lack of comfort at home had almost 2 times higher odds of low QoL in moods and emotions (OR = 1.93; 95% CI: 1.05–3.55) and more than 4 times higher odds in the parent relation and home (OR = 4.21; 95% CI: 2.20–8.03) dimensions.

The last examined group of variables was a group concerning the adolescent’s lifestyle, such as using social media, physical activity and relation with peers. Time spent on social media (instant messengers, social networks) amounting to more than 3 h per week was related to 52% higher odds of low QoL only in the moods and emotions domain (OR = 1.52; 95% CI: 1.08–2.15). Less than 3 h or 3 h per week of physical activity increased the odds of low QoL in a range from 1.5 times to 4.93 times in domains concerning physical and psychological well-being, moods and emotions and social support and peers. Adolescents who reported not spending their free time with friends outside of school were more likely to experience low QoL in five out of ten dimensions: physical well-being (OR: 1.74; 95% CI: 1.04–2.92), psychological well-being (OR: 3.44; 95% CI: 1.94–6.09), self-perception (OR:1.86; 95% CI: 1.09–3.16), social support and peers (OR: 2.93; 95% CI: 1.77–4.86), and social acceptance and bullying (OR: 2.02, 95% CI: 1.18–3.45). The detailed information about the odds ratio of low QoL in KIDSCREEN-52 dimensions for adolescents are presented in Table 3.

## 4. Discussion

In our study, we wanted to indicate determinants of different dimensions of quality of life (QoL) in the fields of demographic, socioeconomic factors, general health, and lifestyle among a sample of adolescents from a southern region of Poland. 

In our study, the quality of life of adolescents in all dimensions was found to be slightly lower than that assessed for the reference European population in the KIDSCREEN project, for which the value of 50 was supposed to be an average level of QoL for each dimension [21], especially for the school environment and the psychological well-being dimensions. The data for all QoL dimensions were categorized according to the guidelines of the authors of the KIDSCREEN-52 [21], and then our study showed that the highest prevalence (frequency) of low QoL among adolescents was observed in the psychological well-being and school environment dimensions, whereas school environment was assessed as the lowest out of all the KIDSCREEN-52 dimensions. In these dimensions, more than 50% of adolescents assessed their QoL as low. 

In our population, most of the adolescents declared good family material standing (85.7%) and received pocket money (90.6%). These results are also confirmed by other studies among adolescents in Poland and Europe using the KIDSCREEN questionnaire. The Polish nationwide study of quality of life among children and adolescents conducted in 2021 among students from the 2nd and 6th grades of elementary school and the 2nd grade of upper secondary school identified that the majority of adolescents assessed their material standing in their family as good—80% declared that they have enough money for current needs and all expenses [22]. In the study conducted by the KIDSCREEN Group among students from seven European countries, more than 80% of adolescents declared medium or high family wealth [16]. 

The majority of young people in our study were satisfied with their health status and appearance. Furthermore, in an international HBSC (Health Behavior in School Age Children) study in 2018 among Polish adolescents aged 11–15, conducted by Mazur et al. [23], indicated that the majority of young people were satisfied with their health status, namely that only 14.2% of the adolescents assessed their health as worse than good [23]. Then, in our population, almost 70% of the adolescents declared being satisfied with their appearance. In turn, in a Spanish study based on HBSC consists of 4531 adolescents aged 13 to 18 years, most of the youth declared medium satisfaction with body image (36.9%); however, this was greater than those who declared low satisfaction with their appearance [24].

In our study, only about 19% of the adolescents declared more than 3 h of physical activity per week (besides physical education classes). Similar observation was found in the study of Wojtyła et al. [25]. In the study conducted in a representative school sample of 12,000 Polish students in upper secondary schools, most of them declared that their main physical activities were only during physical education classes (88%) and going to school (61%) by day. The authors indicated that physical activity among Polish adolescents is not at a high level [25]. In our population sample, almost 88% of the adolescents reported spending their free time with friends outside of school. A similar observation was found in a study conducted by the Polish Foundation for Children and Adolescents in a sample of adolescents aged 13–18. They declared that 60% of them spend their free time with friends every day outside of school [26]. 

The study indicated that sex and school year were significant predictors of QoL. Girls were more likely to report lower QoL than boys in the majority of KIDSCREEN-52 dimensions (physical well-being, psychological well-being, self-perception, autonomy, parent relation and home life, financial resources). This result was confirmed in the other studies, both conducted in the Polish population [5,20] and in different countries [2,10,11,12,13,27,28]. In the Polish study among adolescents from junior high school in Kraków using KIDSCREEN-27, the authors also indicated that girls assessed their QoL as lower than boys in terms of physical and psychological well-being, autonomy and relations with parents [5], whereas a study conducted in Poland among the 8–18 years old (the Polish part of KIDSCREEN project) showed that girls reported lower QoL than boys in physical well-being, moods and emotions, self-perception, and autonomy dimensions [20]. These results are also supported by global studies using the KIDSCREEN-52 instrument, in which boys scored their QoL higher than girls in the majority of dimensions, in particular in psychological well-being, moods and emotions [2,13,14,27,29,30]. 

The lower quality of life among girls might be explained by biological and psychological emotional differences between girls and boys. Biological differences such as earlier puberty and brain development can underpin explanations of lower quality of life in girls [31,32]. Girls experience all the changes in the body (for example, the first menstrual period, increasing outer body fat) and psyche more negatively than boys [2,28]. This might lead to negative body image [33]. Adolescent girls are less disposed to physical activity than adolescent boys, and girls prefer passive leisure time. Gaspar et al. suggested that boys probably achieve higher QoL than girls in the physical well-being dimension due to their physical activity with friends, such as collective games and other activities, in their free time [13]. Girls are marked by lower self-assessment, more frequent depressive symptoms and negative emotions than boys. Additionally, girls are less satisfied with their life [2,33,34], so this might explain their poorer QoL in the psychological well-being and self-perception dimensions. Lower QoL in the context of autonomy and relation with parents might be explained by the discrepancy between the sense of autonomy of adolescents and parental control, which is associated with lower level of well-being. Piko and Hamvai suggested that girls report parental monitoring more often than boys, which is related to lower life satisfaction [17]. In our study, girls experienced poorer QoL in the financial resources dimension. This might be associated with the fact that girls feel a financial deficit in families more strongly than boys, and they do not have enough pocket money for spending [35]. 

Being students in the 3rd grade of junior high school and the 2nd grade of upper secondary school was related to higher odds of low QoL in adolescents (in dimensions relating to physical and psychological well-being, moods and emotions, self-perception and relations with peers and school). It can be suggested that younger pupils assess their QoL as higher than the older ones. A study conducted in a representative school-based sample of Norwegian students aged 8–16 years showed statistically significant differences related to grade in various domains of QoL. Students in the 8th grade reported a decrease in QoL over the six-month follow-up period as compared to those in the 6th grade with regard to the QoL family and school domains and total QoL [36]. A similar observation was found in other studies [13,14]. This can be related to the fact that older adolescents encounter more and more difficult challenges concerning the increasing requirements in school, which may impair QoL among youth.

Our findings also demonstrate that variables concerning general health, such as being dissatisfied with appearance, health status, or being absent at school seldom or often due to illness episodes were significant predictors of QoL in adolescents. The subjective sense of satisfaction with body and appearance is an important factor affecting quality of life in teenagers [37]. The contemporary model of an ideal body promoted by the mass media affects the social expectations of youth and may be a source of dissatisfaction with one’s own body [38]. Griffits et al. found that the reduction in the level of quality of life associated with dissatisfaction with the body was equally pronounced in girls and boys. It concerned the reduction in the quality of life in physical and psychosocial dimensions [11]. In our study, adolescents who reported dissatisfaction with health status had higher odds of low QoL. In an American study, the authors indicated that if adolescents reported ′fair or poor’ health or an increasing number of poor physical health days, the risk of dissatisfaction with life increased [39]. Moreover, experiencing illness episodes and absence at school for this reason may reduce the QoL of adolescents due to worse physical well-being as well as possible difficulties in catching up with the school material. Garcia-Moya et al. showed that absence from school and backlogs in school material may reduce the QoL in youth and, as a result, may impair their health status [40].

Our study also found that socio-economic variables affected the QoL in adolescents. We found that lower (primary/vocational) father’s/caregiver’s education, not receiving pocket money, bad material standing of the family, bad relations with at least one parent/caregiver, and feeling uncomfortable at home significantly decreased the QoL among youth.

Von Rueden et al. found that wealthiness of the family and the level of parents’ education have a significant impact on the QoL in childhood and adolescence, whereby the family’s wealthiness was a stronger predictor of the quality of life. Primary or secondary parental education was related to lower child QoL concerning physical and psychological well-being, moods and emotions, social acceptance, and financial resources. Among the adolescents, only physical well-being was related to the parental education. Children from families with low or medium financial resources had higher odds of poor QoL in the following dimensions: physical well-being, parent relations and home life, and financial resources in comparison to their peers from wealthier families. In adolescents, low or middle financial resources in family were a risk factor for low QoL in all KIDSCREEN-52 dimensions. Lower financial resources in a family may also suggest that a teenager does not receive any pocket money from parents/caregivers [16]. Good relations with parents/caregivers and as a consequence a feeling of comfort at home are also important predictors of QoL, so the lack of closer relations mean that a teenager does not feel comfortable at home, which may reduce QoL. Piko and Hamvai found that parents and the relations with them play a key role in the achievement of well-being among adolescents, especially joint meals and talking about problems with parents [17]. We also confirmed that spending more than 3 h on social media per week, a low level of physical activity, and not spending free time with friends outside of school significantly increased the odds of low QoL in adolescents.

Frequent use of electronic media may be associated with poorer behavior, health status, and QoL in adolescents. A relationship between the time spent using the computer and on the Internet and mental discomfort was observed [41]. Intensive use of social media may be associated with more frequent psychosocial problems (e.g., anxiety and depression, abnormal thinking) [42,43]. Abnormal thinking involves a disturbance in how thoughts are organized and expressed. It causes disorganized thinking and leads to people expressing themselves in unusual ways when speaking or writing [44]. Furthermore, frequent use of social media may be related to low physical activity. Studies indicate that low level of physical activity is related to poorer QoL in physical fitness, emotions, and general health domains [45], whereas a higher level of physical activity is related to higher QoL [46,47]. This may be explained by the fact that regular physical activity improves psychological well-being, so consequently it may improve the QoL.

Relations with peers are a very important element of adolescents’ life, which significantly affects the QoL of young people. Moreover, Piko and Hamvai showed that helpful friends and relationships with them were important predictors of well-being in girls, increasing the QoL among them [17].

The international survey—HBSC (Health Behavior in School-aged Children)—conducted in Poland in 2010 among students of the 1st and the 3rd grade of junior high schools relevant to relations with friends and their QoL showed that better relations with friends was significantly associated with a higher QoL in students from junior high schools; however, this relationship was more clearly visible in boys. A lack of friendly relationships may lead to a reduction in QoL as well as increase the intensity of emotional and social problems among adolescents [48]. Knowledge about predictors of QoL among youth is especially important for preventive medicine and public health, but it is also important for specialists, teachers, psychologists and other who meet and work with adolescents. Knowledge about predictors of QoL can help in the identification of reasons for low QoL and the development of preventive actions for this age group.

Schools should also provide opportunities to improve adolescents’ QoL. Health promotion and intervention programs that aim to strengthen psychosocial well-being, especially self-image, should be developed for adolescents [7]. This will help us to better understand perceived health in children and adolescents and identify populations at risk [49]. It might be considered both in the development of social policy and in initiatives for adolescents. Preventive actions for adolescents should be popularized in public service announcement (i.e., promoting physical activity among adolescents) and through schools or non-governmental organizations [22]. The results of our study might be important for professionals working with adolescents (i.e., psychologists).

### Limitations and Strengths of the Present Study

The present study has some disadvantages. Firstly, our study is cross-sectional, so it does not enable the assessment of the causality between the potential determinants of QoL and low QoL. Then, it should be considered that despite the fact that information about the study was given to parents during periodical teacher–parent meetings, we did not receive any information (either approval or refusal to participate) from 33% of the adolescents. It should be also emphasized that there were restrictions concerning the respondents’ errors (respondent gave a reply contrary to the state of fact). Due to the cross-sectional design of our study, it is not usual to describe the associations in the form of relative risk, because there is no time sequence analyzed in this design, so we did not use Poisson regression. We would rather use a logistic regression model.

Some strengths should be mentioned as well. The QoL of adolescents was assessed using the KIDSCREEN-52 questionnaire prepared as a multinational instrument and validated both internationally and in Poland. In addition, the sample size was big enough to receive reliable estimates and ensure that the results were representative of adolescents in the southern part of Poland. This study provides information about the potential determinants of the quality of life of the adolescent population. According to our knowledge, the present study is the first of this kind in Poland and in the Małopolska region.

## 5. Conclusions

In conclusion, our study reveals that different risk factors were related to low QoL in different dimensions: female sex was a risk factor for physical well-being, psychological well-being, self-perception, autonomy, parent relation and home life, and financial resources; higher school year was for physical well-being, psychological well-being, moods and emotions (2nd grade of upper secondary school only), self-perception, social support and peers, and school environment; and dissatisfaction with appearance was for physical well-being, psychological well-being, moods and emotions, self-perception, school environment, and social acceptance and bullying.

## Figures and Tables

**Table 1:** Characteristics of the sample in adolescents aged 12–18 years (*n* = 804).

Variable	*N* (%)
Sex	
Girls	393 (48.9)
Boys	411 (51.1)
School year	
1st grade of junior high school	186 (23.1)
3rd grade of junior high school	152 (18.9)
2nd grade of upper secondary school	466 (58)
Satisfaction with health status (2)	
No	121 (15.1)
Yes	681 (84.9)
Satisfaction with appearance (1)	
No	252 (31.4)
Yes	551 (68.6)
Absence at school due to illness episodes (2)	
never/seldom (<1 × /3 months)	687 (85.7)
often (>1 × /2 months)	115 (14.3)
Father’s/Caregiver’s education (13) *	
primary/vocational	459 (59.6)
secondary/higher	311 (40.4)
Receiving pocket money (5)	
No	75 (9.4)
Yes	724 (90.6)
Good family material standing (5)	
No	114 (14.3)
Yes	685 (85.7)
Good relations with at least one parent/caregiver (1)	
No	63 (7.9)
Yes	740 (92.1)
Feeling comfortable at home	
No	81 (10.1)
Yes	723 (89.9)
Time for social media (instant messengers, social networks) per week (14) **	
≤3 h	516 (65.4)
>3 h	273 (34.6)
Time for physical activity per week (besides physical education classes) (5)	
≤3 h	646 (80.9)
>3 h	153 (19.1)
Spending free time with friends besides school (4)	
No	99 (12.4)
Yes	701 (87.6)

* adolescents without living father/caregiver (*n* = 21) not included. ** adolescents who did not use social media (*n* = 1) not included, missing data presented in brackets.

**Table 2:** The quality of life in adolescents in 10 KIDSCREEN-52 dimensions (T-scores).

The KIDSCREEN-52 Dimension	Total *n* = 804		
	Percentiles		
	25	50	75
Physical Well-being	40.45	46.81	51.17
Psychological Well-being	36.91	43.25	49.34
Moods and Emotions	40.00	45.44	54.02
Self-Perception	40.52	44.58	49.76
Autonomy	39.98	45.18	50.77
Parent Relation and Home Life	39.69	45.72	54.65
Financial Resources	41.92	49.28	56.35
Social Support and Peers	39.49	43.60	48.35
School Environment	38.15	42.35	46.94
Social Acceptance and Bullying	42.20	58.85	58.85

**Table 3:** The odds ratio of low QoL in KIDSCREEN-52 dimensions for adolescents.

	Physical Well-Being	Psychological Well-Being	Moods and Emotions	Self-Perception	Autonomy	Parent Relation and Home Life	Financial Resources	Social Support and Peers	School Environment	Social Acceptance and Bullying
	OR (95% CI)	OR (95% CI)	OR (95% CI)	OR (95% CI)	OR (95% CI)	OR (95% CI)	OR (95% CI)	OR (95% CI)	OR (95% CI)	OR (95% CI)
Sex										
boys	1.00 (Ref.)	1.00 (Ref.)	1.00 (Ref.)	1.00 (Ref.)	1.00 (Ref.)	1.00 (Ref.)	1.00 (Ref.)	1.00 (Ref.)	1.00 (Ref.)	1.00 (Ref.)
girls	2.59 (1.80–3.71)	1.84 (1.30–2.60)	1.32 (0.94–1.87)	1.66 (1.17–2.36)	3.47 (2.44–4.91)	1.50 (1.04 –2.15)	1.53 (1.02–2.27)	1.16 (0.83–1.62)	1.14 (0.82–1.59)	0.76 (0.49–1.19)
School Year										
1st grade of junior high school	1.00 (Ref.)	1.00 (Ref.)	1.00 (Ref.)	1.00 (Ref.)	1.00 (Ref.)	1.00 (Ref.)	1.00 (Ref.)	1.00 (Ref.)	1.00 (Ref.)	1.00 (Ref.)
3rd grade of junior high school	2.13 (1.23–3.69)	2.11 (1.26–3.55)	1.53 (0.91–2.58)	2.41 (1.43–4.06)	1.38 (0.84–2.28)	1.14 (0.67–1.95)	1.56 (0.88–2.75)	2.56 (1.57–4.19)	2.04 (1.26–3.29)	0.73 (0.40–1.33)
2nd grade of upper secondary school	3.14 (1.98–4.98)	1.96 (1.29–2.97)	1.89 (1.24–2.89)	1.58 (1.03–2.42)	1.40 (0.93–2.10)	1.17 (0.76–1.82)	1.28 (0.80–2.05)	2.13 (1.42–3.19)	2.56 (1.72–3.79)	0.59 (0.36–0.96)
Satisfaction with Health Status										
yes	1.00 (Ref.)	1.00 (Ref.)	1.00 (Ref.)	1.00 (Ref.)	1.00 (Ref.)	1.00 (Ref.)	1.00 (Ref.)	1.00 (Ref.)	1.00 (Ref.)	1.00 (Ref.)
no	1.69 (1.02–2.80)	2.07 (1.20–3.58)	1.25 (0.77–2.04)	1.57 (0.92–2.66)	0.98 (0.61–1.61)	1.72 (1.05–2.82)	1.14 (0.66–1.94)	0.99 (0.62–1.59)	1.23 (0.75–2.01)	1.26 (0.72–2.19)
Satisfaction with Appearance										
yes	1.00 (Ref.)	1.00 (Ref.)	1.00 (Ref.)	1.00 (Ref.)	1.00 (Ref.)	1.00 (Ref.)	1.00 (Ref.)	1.00 (Ref.)	1.00 (Ref.)	1.00 (Ref.)
no	1.80 (1.23–2.62)	2.07 (1.41–3.02)	2.42 (1.69–3.48)	5.22 (3.54–7.70)	1.26 (0.88–1.82)	1.44 (0.98–2.10)	1.48 (0.99–2.23)	1.31 (0.92–1.88)	1.90 (1.33–2.73)	2.01 (1.29–3.14)
Absence at School due to Illness Episodes										
never/seldom (<1 × /3 months)	1.00 (Ref.)	1.00 (Ref.)	1.00 (Ref.)	1.00 (Ref.)	1.00 (Ref.)	1.00 (Ref.)	1.00 (Ref.)	1.00 (Ref.)	1.00 (Ref.)	1.00 (Ref.)
often (<1 × /2 months)	2.47 (1.48–4.13)	1.04 (0.63–1.71)	1.13 (0.69–1.84)	1.10 (0.67–1.82)	1.08 (0.66–1.75)	1.25 (0.76–2.07)	0.57 (0.32–1.03)	1.33 (0.84–12.12)	1.78 (1.10–2.89)	1.65 (0.93–12.94)
Father’s/Caregiver’s Education										
secondary/higher	1.00 (Ref.)	1.00 (Ref.)	1.00 (Ref.)	1.00 (Ref.)	1.00 (Ref.)	1.00 (Ref.)	1.00 (Ref.)	1.00 (Ref.)	1.00 (Ref.)	1.00 (Ref.)
primary/vocational	1.12 (0.78–1.59)	1.00 (0.71–1.40)	1.19 (0.85–1.67)	1.49 (1.06–2.10)	0.98 (0.70– 1.37)	1.43 (1.00–2.04)	1.42 (0.97–2.09)	1.01 (0.74–1.39)	1.05 (0.77–1.44)	0.97 (0.64–1.48)
Receiving Pocket Money										
yes	1.00 (Ref.)	1.00 (Ref.)	1.00 (Ref.)	1.00 (Ref.)	1.00 (Ref.)	1.00 (Ref.)	1.00 (Ref.)	1.00 (Ref.)	1.00 (Ref.)	1.00 (Ref.)
no	1.39 (0.76–2.53)	1.64 (0.86–3.13)	1.82 (1.01–3.28)	0.94 (0.51–1.75)	2.28 (1.24–4.19)	1.57 (0.87–2.84)	6.43 (3.42–12.11)	2.25 (1.27–3.98)	1.67 (0.94–2.97)	1.84 (1.00–3.39)
Good Material Standing in Family										
yes	1.00 (Ref.)	1.00 (Ref.)	1.00 (Ref.)	1.00 (Ref.)	1.00 (Ref.)	1.00 (Ref.)	1.00 (Ref.)	1.00 (Ref.)	1.00 (Ref.)	1.00 (Ref.)
no	1.23 (0.74–2.06)	2.84 (1.58–5.09)	1.86 (1.13–3.07)	1.38 (0.81–2.36)	2.64 (1.57–4.44)	1.60 (0.97–2.66)	5.34 (3.18–8.97)	1.05 (0.65–1.70)	1.36 (0.83–2.23)	1.23 (0.69–2.19)
Good Relations with at Least One Parent/Caregiver										
yes	1.00 (Ref.)	1.00 (Ref.)	1.00 (Ref.)	1.00 (Ref.)	1.00 (Ref.)	1.00 (Ref.)	1.00 (Ref.)	1.00 (Ref.)	1.00 (Ref.)	1.00 (Ref.)
no	1.85 (0.95–3.61)	2.37 (1.13–4.97)	2.10 (1.09–4.04)	1.83 (0.91–3.67)	1.88 (0.98–3.62)	5.67 (2.73–11.81)	2.00 (1.01–3.94)	1.98 (1.06–3.71)	1.93 (1.00–3.70)	1.46 (0.73–2.95)
Feeling Comfortable at Home										
yes	1.00 (Ref.)	1.00 (Ref.)	1.00 (Ref.)	1.00 (Ref.)	1.00 (Ref.)	1.00 (Ref.)	1.00 (Ref.)	1.00 (Ref.)	1.00 (Ref.)	1.00 (Ref.)
no	1.39 (0.75–2.60)	1.47 (0.76–2.86)	1.93 (1.05–3.55)	1.74 (0.91–3.35)	1.30 (0.70–2.40)	4.21 (2.20–8.03)	0.83 (0.42–1.62)	1.05 (0.58–1.88)	0.96 (0.53–1.73)	1.22 (0.62–2.38)
Time for Social Media (Instant Messengers, Social Networks) Per Week										
≤3 h	1.00 (Ref.)	1.00 (Ref.)	1.00 (Ref.)	1.00 (Ref.)	1.00 (Ref.)	1.00 (Ref.)	1.00 (Ref.)	1.00 (Ref.)	1.00 (Ref.)	1.00 (Ref.)
>3 h	1.02 (0.71–1.46)	0.87 (0.61–1.23)	1.52 (1.08–2.15)	1.34 (0.94–1.91)	0.50 (0.35–0.71)	1.22 (0.85–1.75)	0.87 (0.58–1.30)	0.65 (0.47–0.91)	0.91 (0.65–1.26)	1.17 (0.76–1.81)
Time for Physical Activity Per Week (Besides Physical Education Classes)										
>3 h	1.00 (Ref.)	1.00 (Ref.)	1.00 (Ref.)	1.00 (Ref.)	1.00 (Ref.)	1.00 (Ref.)	1.00 (Ref.)	1.00 (Ref.)	1.00 (Ref.)	1.00 (Ref.)
≤3 h	4.93 (2.82–8.61)	1.93 (1.25–2.96)	1.73 (1.11–2.68)	1.10 (0.72–1.69)	1.38 (0.90–2.11)	1.51 (0.95–2.41)	1.09 (0.67–1.78)	1.50 (1.01–2.24)	0.94 (0.64–1.40)	1.19 (0.68–2.06)
Spending Free Time with Friends Outside of School										
yes	1.00 (Ref.)	1.00 (Ref.)	1.00 (Ref.)	1.00 (Ref.)	1.00 (Ref.)	1.00 (Ref.)	1.00 (Ref.)	1.00 (Ref.)	1.00 (Ref.)	1.00 (Ref.)
no	1.74 (1.04–2.92)	3.44 (1.94–6.09)	1.60 (0.98–2.64)	1.86 (1.09–3.16)	1.34 (0.82–2.20)	1.31 (0.79–2.19)	1.31 (0.77–2.24)	2.93 (1.77–4.86)	1.57 (0.96–2.57)	2.02 (1.18–3.45)

OR—odds ratio, CI—confidence interval, Ref. – reference group.

## Data Availability

Not applicable.

## Abbreviations

QoL	Quality of life
WHO	The World Health Organization
SD	Standard deviation
OR	Odds ratio
CI	Confidence interval
Ref.	Reference group
